# Metagenomic Analysis of a Southern Maritime Antarctic Soil

**DOI:** 10.3389/fmicb.2012.00403

**Published:** 2012-12-05

**Authors:** David A. Pearce, Kevin K. Newsham, Michael A. S. Thorne, Leo Calvo-Bado, Martin Krsek, Paris Laskaris, Andy Hodson, Elizabeth M. Wellington

**Affiliations:** ^1^Ecosystems Programme, Natural Environment Research Council, British Antarctic SurveyCambridge, UK; ^2^School of Life Sciences, University of WarwickCoventry, UK; ^3^Department of Geography, University of SheffieldSheffield, UK

**Keywords:** Antarctica, bacteria, metagenomics, polar, soil, 454 pyrosequencing

## Abstract

Our current understanding of Antarctic soils is derived from direct culture on selective media, biodiversity studies based on clone library construction and analysis, quantitative PCR amplification of specific gene sequences and the application of generic microarrays for microbial community analysis. Here, we investigated the biodiversity and functional potential of a soil community at Mars Oasis on Alexander Island in the southern Maritime Antarctic, by applying 454 pyrosequencing technology to a metagenomic library constructed from soil genomic DNA. The results suggest that the commonly cited range of phylotypes used in clone library construction and analysis of 78–730 OTUs (de-replicated to 30–140) provides low coverage of the major groups present (∼5%). The vast majority of functional genes (>77%) were for structure, carbohydrate metabolism, and DNA/RNA processing and modification. This study suggests that prokaryotic diversity in Antarctic terrestrial environments appears to be limited at the generic level, with *Proteobacteria*, *Actinobacteria* being common. Cyanobacteria were surprisingly under-represented at 3.4% of sequences, although ∼1% of the genes identified were involved in CO_2_ fixation. At the sequence level there appeared to be much greater heterogeneity, and this might be due to high divergence within the relatively restricted lineages which have successfully colonized Antarctic terrestrial environments.

## Introduction

Antarctic soils have fascinated microbiologists throughout the last century. Expeditions to the continent as far back as 1901 collected soils specifically for bacteriological study. The results of these studies, and later twentieth century research, indicated that Antarctic soils typically harbor low numbers of bacterial taxa (Wynn-Williams, 1996). It is thought that this low taxonomic diversity is associated with increasingly severe environmental conditions, such as restricted water and nutrient availability, and frequent freeze-thaw cycling in summer. These environmental factors also increase in severity with progression from the island archipelagos of the Scotia arc and northern Antarctic Peninsula southwards to the Antarctic continent (Convey, 2001). However, the evidence for this restricted taxonomic diversity has been largely based on data from culture-based studies (e.g., Wynn-Williams, 1983) and, more recently, on data from clone libraries (Table 1). These studies have typically only provided levels of coverage of 0.50–0.80 (but see Aislabie et al., 2009), and all have recorded similar levels of biodiversity (at ∼30–140 OTUs), all of which could be considered to be relatively low when compared with soils sampled from temperate or tropical environments (Fierer et al., 2003, 2007; Fierer and Jackson, 2006). Many of the dominant bacterial taxa identified by previous studies of Antarctic soil fall into similar taxonomic categories (Table 2). However, in recent years, with the advent of metagenomic technology (Handelsman et al., 1998; Hugenholz et al., 1998; Eisen, 2007; Delmont et al., 2011) it is now possible to make a more comprehensive assessment of the scope of the microbial biodiversity present in these soils, and even to determine some of the potential geochemical functions of these microbial communities.

**Table 1:** **Summary data from a selection of previous studies of Antarctic soil microbial diversity**.

Reference	Latitude and longitude (place name)	T	S	R	% D	Shannon–Weaver DI	Coverage	Richness
Aislabie et al. (2006)	S 77° 25′E 163° 41′ (Marble Point)	728	33	52–85	44–56	2.65–3.95	0.50–0.52	n/a
	S 77° 31′ E 161° 52′ (Bull Pass)	n/a	n/a	29–47	82–85	2.53–3.19	0.81–0.83	n/a
	S 77° 31′ E 161° 40′ (Lake Vanda)	n/a	n/a	47–61	67–69	3.27–3.32	0.70–0.70	n/a
Saul et al. (2005)	S 77° 50′ E 166° 45′ (Scott Base)	522	62	56	n/a	3.70–3.76	n/a	n/a
Aislabie et al. (2008)	S 77° 55′ E 166° 45′ (Scott Base)	155	n/a	45–51	11–18	n/a	0.52–0.57	46–182
	S 77° 25′ E 163° 41′ (Marble Point)	131	n/a	47–85	4–12	n/a	0.50	n/a
	S 77° 31′ E 161° 52′ (Bull Pass)	236	n/a	29–47	23–24	n/a	0.78–0.83	n/a
	S 77° 31′ E 161° 40′ (Lake Vanda)	211	n/a	47–49	16–17	n/a	0.63–0.70	n/a
	S 77° 19′ E 170° 13′ (Cape Hallett)	173	n/a	26	19–27	n/a	0.77–0.80	n/a
Aislabie et al. (2009)	S 77° 19′ E 170° 13′ (Cape Hallett)	580	52	27–57	29–76	n/a	0.45–0.78	63–256
	S 77° 13′ E 166° 26′ (Cape Bird)	168	11	4–19	78–99	n/a	0.85–0.99	5–36
Smith et al. (2006)	S 78° 05′ E 165° 53′ (PENP)	181	61	n/a	n/a	1.598	0.73	n/a
	S 78° 06′ E 165° 49′ (MVG)	n/a	n/a	n/a	n/a	1.331	0.64	n/a
	S 78° 01′ E 165° 33′ (BIS)	n/a	n/a	n/a	n/a	1.238	0.56	n/a
Taton et al. (2003)	S 77° 37′ E 163° 07′ (Lake Fryxell)	78	16	15	n/a	2.88	0.79	n/a
Niederberger et al. (2008)	S 72° 22′ E 169° 53′ (Luther Vale)	323	323	n/a	n/a	3.32–4.04	n/a	n/a
Yergeau et al. (2007)	54° 15′ S, 36° 30′ W (South Georgia)	178	2111	130	n/a	n/a	n/a	470
	60° 43′ S, 45° 38′ W (Signy Island)	174	n/a	128	n/a	n/a	n/a	420
	67° 34′ S, 68° 08′ W (Anchorage Island)	154	n/a	100	n/a	n/a	n/a	430
	71° 19′ S, 68° 18′ W (Fossil Bluff)	183	n/a	60	n/a	n/a	n/a	180
	71° 53′ S, 68° 15′ W (Mars Oasis)	168	n/a	138	n/a	n/a	n/a	460
	72° 03′ S, 68° 31′ W (Coal Nunatak)	187	n/a	40	n/a	n/a	n/a	100
	78° 26′ S, 85° 60′ W (Ellsworth Mountains)	170	n/a	98	n/a	n/a	n/a	270
Yergeau et al. (2009)	60° 43′ S, 45° 38′ W (Signy Island)	320	n/a	n/a	n/a	n/a	n/a	420
	67° 34′ S, 68° 08′ W (Anchorage Island)	367	n/a	n/a	n/a	n/a	n/a	430
	71° 19′ S, 68° 18′ W (Fossil Bluff)	107	n/a	n/a	n/a	n/a	n/a	180
	72° 03′ S, 68° 31′ W (Coal Nunatak)	160	n/a	n/a	n/a	n/a	n/a	100

*T, total number of clones; S, number of sequences deposited; R, number of ribotype or phylotype patterns; % D, clones assigned to dominant ribotype (percentage); DI, diversity Index; n/a, not available*.

**Table 2:** **Many of the dominant bacterial taxa identified by previous studies on Antarctic soil fall into similar taxonomic categories**.

*Rubrobacter*, *Arthrobacter*, *Acidobacteria*, *Oscillatoria*, *Phormidium*, *Deinococcus*, *Sphingomonas*, *Bacteroides*, *Brevundimonas*, *Chloroflexus*, *Hymenobacter*, *Leptolyngbya*, *Nostoc*, *Pseudonocardia*, *Psychrobacter*, *Rhodococcus*, *Synechococcus*, *Actinobacteria*, *Anabaena*, *Cytophaga*, *Fervidobacterium*, *Friedmanniella*, *Microcoleus*, *Microcystis*, *Nitrosospira*, *Pseudomonas*, *Sphingobacterium*, *Sporosarcosina*, and *Xanthomonas*

Antarctic soils are of particular interest because chemical analyses has shown that they are relatively low in nutrient content (Lawley et al., 2004; Newsham et al., 2010). This can lead to strong gradients in physicochemical parameters at a wide range of spatial scales; of the order of meters (Chong et al., 2010), kilometers (Chong et al., 2011), or hundreds of kilometers (Yergeau et al., 2007). Antarctic soils also provide extremely good early indications of the potential effects of environmental change. The Antarctic Peninsula, for example, is warming three times faster than the global average (Turner et al., 2005).

Unsurprisingly, an increasing number of studies are beginning to show that the microbial biodiversity associated with these environmental gradients could be much larger than was once thought. Mars Oasis was chosen for this study as preliminary data already exist for this site. It has been suggested to be a potential biodiversity hotspot (Yergeau et al., 2007) and it has unique soil chemistry when compared to the surrounding area (Chong et al., 2011). It is also geographically isolated, being situated 1,000 km from South America on the south-eastern coast of Alexander Island in the southern Maritime Antarctic, and is isolated by the Antarctic Circumpolar current and prevailing wind direction from the continental interior. Through studies of aerobiological transfer at Rothera (Hughes et al., 2004) and Halley (Pearce et al., 2010) we have some idea of the type of colonist arriving via aerial transfer, and there is relatively little wildlife or human impact at the site. One such study (Newsham et al., 2010) showed no difference between microbial biodiversity across two different parts of the same site at the 97% sequence homology level. However, the effects of alignment quality, distance calculation method, sequence filtering, and region on the analysis of 16S rRNA gene can all influence biodiversity estimates (Schloss, 2010). A re-analysis of data from this study, showed that the biodiversity could be different at each of the two study sites examined depending upon the specific criteria used for sequence differentiation. Hence the site may contain a higher diversity than that shown by clone library analysis alone.

Here, we report the biodiversity and functional potential of the soil community at Mars Oasis, based on the application of 454 pyrosequencing technology to a metagenomic library. It is important to recognize that all techniques in molecular biology impose some degree of bias or selection, and indeed numerous studies have investigated new methods to improve extraction, purification, amplification, and quantification of DNA from soils. In addition, comparative studies have been performed to analyze the efficiency of methods for extraction and purification of soil DNA recovered, and there are a number of excellent reviews in the literature which consider this topic in some detail (for example, Wintzingerode et al., 1997; Frostegård et al., 1999; Courtois et al., 2001; Martin-Laurent et al., 2001; Feinstein et al., 2009; Delmont et al., 2011; Mahmoudi et al., 2011). For this reason, we do not attempt to provide a systematic analysis of the differentiation based on 16S rDNA. We rather highlight that a polyphasic approach can significantly increase the apparent diversity present and to focus on the relative magnitude and direction of the difference rather than absolute values. This is particularly important for Antarctic soils now, where the total biodiversity was believed to be limited. This view is changing. So the aim of this study was to gain a more comprehensive understanding of the taxonomic diversity of bacteria present in the soil and to determine an initial frequency distribution of potential functional genes. By combining the latter data with analyses of the chemistry of runoff and lake water, we also aimed to try to gain some preliminary insight into the main elements being utilized and cycled by the soil microbial community.

## Materials and Methods

### Site description and sampling

Mars Oasis consists of an upper and lower terrace formed from shales. The soil used in this study was collected from the lower terrace, which is situated on a moraine ridge formed by contact between the George VI ice-shelf and Alexander Island. The lower terrace consists of areas of till, fluvial, and lacustrine sediments, with streams and ponds forming during the austral spring and summer. Restricted stands of bryophytes occur on the lower terrace close to meltwater streams and ponds. Lichens are sparse at the site, from which higher animals, including seals and nesting birds, are absent. Mean monthly air and soil (20 mm depth) temperatures at Mars Oasis vary between 1 and 6°C in January and −20 and −15°C in June, respectively. Snow depth at the site is typically ∼2 m in winter, but snow ablates rapidly, usually in November, coinciding with a marked rise in soil water content close to the melt water ponds at the lower site (H. J. Peat, personal communication).

**Figure 1:**
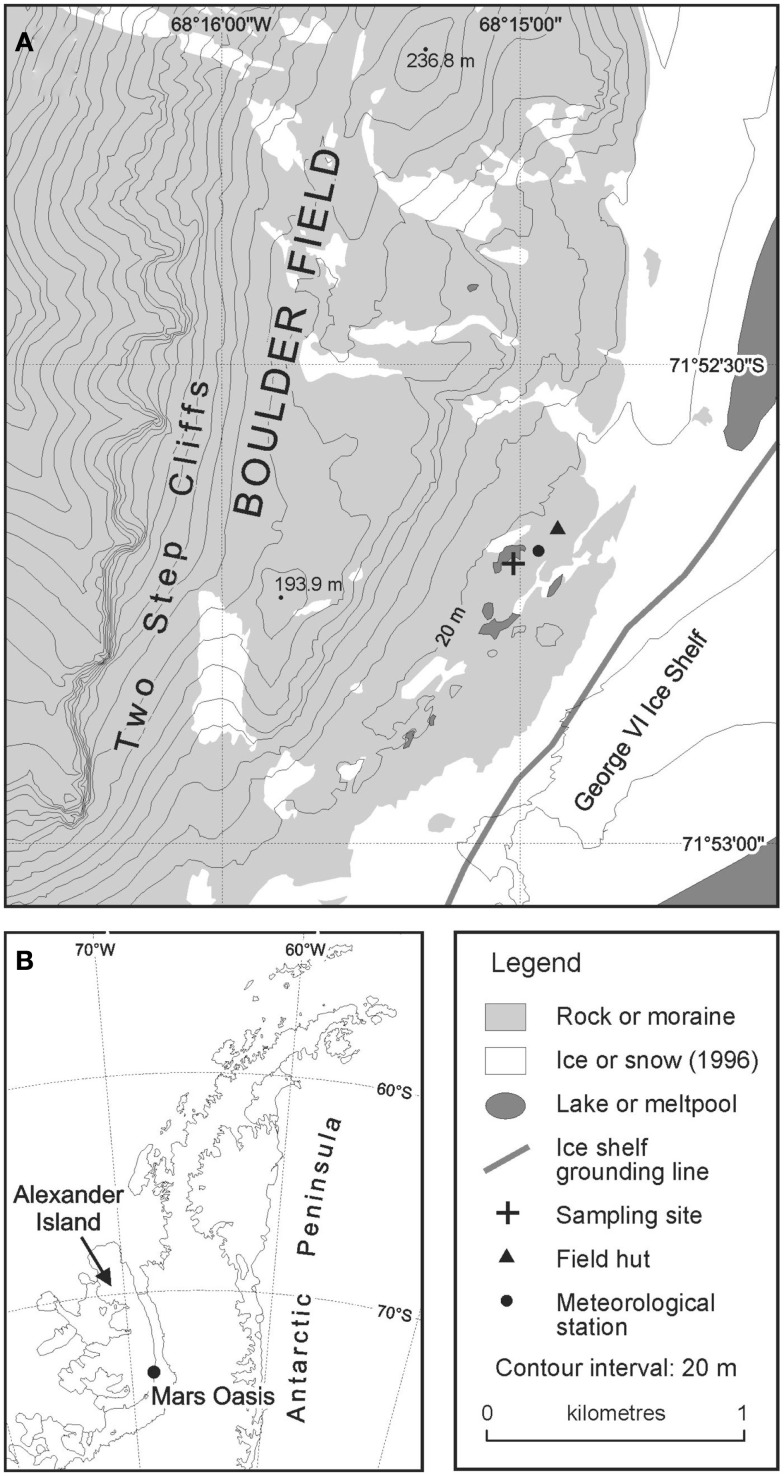
**Location of Mars Oasis (A) on the Antarctic Peninsula (B)**.

The site was accessed from Rothera Research Station on Adelaide Island by fixed-wing aircraft fitted with skis in December 2004. Samples of moraine soil were collected from an area of level ground at the south-eastern margin of a permanent meltwater pool (71°52.6960′ S; 68°14.9879′ W; Figure 1). The soil was collected by inserting four sterile Vacutainers, with their lids removed, to a depth of 5 cm into the soil. Bryophytes were absent from this soil. The lids of the Vacutainers were replaced and the samples placed into re-sealable polythene bags, which were packed in ice in an insulated box. The soils were returned the following day to Rothera Research Station and were frozen at −20°C, prior to their return to the UK at the same temperature.

### Metagenomic library construction

The four soil samples (75 g each) were each suspended in 1% SDS solution (25 ml), to which 0.05 g of glucanex and glucanase had been added. The suspensions were vortexed for a few seconds and then incubated at 37°C for 4 h. They were cooled and filtered (1 mm) and 250 μg of RNAase was added to each solution. The solutions were centrifuged six times at 7,500 rpm for 15 min. After each centrifuge run, the supernatant (12 ml) was decanted. Three molar sodium acetate at pH 7.0 (1.2 ml) and ethanol (26.4 ml) was added to each aliquot of the supernatant, which was centrifuged at 10,000 *g* for 10 min. The pellet of DNA was drained and dried for several minutes. TE buffer (0.1 ml) was added to each pellet, which was then incubated at 4°C for 16 h. All of the TE buffer solutions were combined (∼240 μl), mixed with an equal volume of loading buffer, and ran out in a large-welled 1% low melting point agarose gel (Sigma-Aldrich) at 20 V overnight. The gel ran for 48 h, after which a block of agarose containing the target DNA, which had advanced 14–17 mm, was excised from the gel with a sterile scalpel. A size standard was used to select the region of the gel containing 35–45 kbp fragments. The agarose containing the target nucleic acids was then kept at 4°C for 60 h.

The agarose was digested in Gelase according to the manufacturers’ instructions and enzymes were then denatured by heating to 60°C for 30 s. DNA was precipitated in three separate aliquots and was re-suspended in TE buffer (4.5 μl). The concentration of DNA (3 × 20 ng μl^−1^ aliquots) was determined by running against known standards in 1.5% agarose gels (1 h at 120 V). The DNA was then end-repaired by combining aliquots (12 μl) on ice with 10 × end-repair buffer (1.85 μl), 2.5 mM dNTP mix (1.85 μl), 10 mM ATP (1.85 μl), and end-repair enzyme mix (0.92 μl). The mixture was incubated at room temperature for 45 min and then at 70°C for 10 min. The DNA, consisting of a solution containing 240 ng of DNA, was then ligated into the pEpiFOS-5 fosmid vector (EpiCentre, Madison, WI, USA) by combining it with sterile water (2 μl), 10 × fast link ligation buffer (3 μl), 10 mM ATP solution (3 μl), fastlink ligase (3 μl), and vector (1 μl). The ligation reaction was then incubated at 4°C for 7 days.

The ligation mix was heated to 70°C for 10 min and the fosmid clones were packaged into lambda phages using MaxPlax lambda packaging extracts according to the manufacturer’s instructions (Epicentre, Madison, WI, USA). This process yielded three aliquots (1.025 ml) of cloned cells. The packaged library was transduced into *E. coli* EPI-100, and *E. coli* transformants were selected on LB agar supplemented with 12.5 μg ml^−1^ chloramphenicol. After determining the number of viable cells present, aliquots (200 μl) were spread onto dry Luria broth (100 ml) containing 12.5 μg ml^−1^ chloramphenicol in 47 Petri dishes (150 mm diameter). Chloroformed phage (0.5 ml) was added to EPIFOS cells (5 ml, OD 1.0) and were incubated at 37°C for 20 min. Aliquots (100 μl) were added to each plate, spread, and grown at 37°C for 17 h. Colonies were picked into individual wells of 96 well plates containing Luria broth with 12.5 μg ml^−1^ chloramphenicol (40 μl). The plates were incubated at 37°C for 17 h before sterile glycerol (10% v/v) was added to each well and the plates covered with plastic seals prior to storage at −80°C. All procedures described above took place under a sterile hood.

Quality control was established by end sequencing 20 random fosmids using pEpiFOS^™^-5 forward and reverse end sequencing primers to ensure environmental DNA had been successfully incorporated, from microorganisms that one might expect to find in this extreme environment. One full fosmid was also sequenced. Primers were used to identify specific sequences from the fosmid library. The metagenomic library was screened using a range of primers for viral (Cyanophage CPS4GC, CPS5 Fuller et al., 1998; Wilson et al., 1999 and Phycodnaviridae AVS1, AVS2 Chen and Suttle, 1995), fungal (ITS1F/ITS4F; White et al., 1990; Gardes and Bruns, 1993), phosphonate (Gilbert et al., 2009), and N cycling (nosZ-F/nosZ-R, nirS1F/nirS6R, and nifHF/nifHRb; Thröback et al., 2004; Rösche and Bothe, 2005) genes. A selection of *E. coli* cells containing fosmids were screened for antibiotic production.

Cells from 25 plates (10% of the total) were combined (to favor depth of sequencing rather than coverage) and cultured in Luria broth with 12.5 μg ml^−1^ chloramphenicol overnight in a shaking incubator at 37°C until an OD of 0.8 was obtained. The cells were centrifuge-concentrated and used to construct a 10,000 Gbp metagenomic library for 454 pyrosequencing. Fosmids were extracted from *E. coli* cells using the QIAGEN Plasmid Midi Kit (QIAGEN Plasmid Midi Kit, Cat. No. 12145. QIAGEN) and then treated with ATP-dependent Exo-nuclease (Plasmid-SafeTM ATP-Dependent Dnase, 10 Uμl^−1^ 10,000 U, Cat. No. E3110K, Epicentre). Extracts were sequenced by Macrogen (South Korea) according to the emPCR Method Manual – Lib-L MV (Anon, 2009a) and the Sequencing Method Manual (Anon, 2009b).

### Mars oasis clone library re-analysis

In clone library based studies, it is common to de-replicate samples through RFLP, or to assign sequences to groups with a predetermined sequence similarity (commonly 97%), for the purposes of comparison across different samples, studies, or environmental gradients. The consequence of this approach is a potential underestimate of the total sequence diversity present in any given sample. To estimate the magnitude of this uncertainty, we selected 21 groupings of OTUs derived from a Mars Oasis clone library study (Newsham et al., 2010) and independently aligned them in CLUSTALW, to determine the levels of variation or similarity within each designated group. In the original study, PCR products were aligned in ClustalW and vector sequences removed. Initially these sequences were grouped according to gross similarity by aligning all sequences in Clustal and generating a single average distance tree based on percentage identities. Groups of sequences and any ungrouped sequences were then analyzed as separate data sets. In this re-analysis, this step was modified so that sequences within each group were only retained in that group if they had ≥97% sequence similarity to other members of the same grouping.

### Chemistry

Samples of runoff, lake water, sediment pore water from lake margins and snow were collected in December 2007. The samples were filtered immediately in the field (1 μ m) and then frozen. Sub-samples were stored at ∼1°C in the dark for ∼10 days until pH and ${\text{HCO}}_{3}^{-}$ analyses (alkalinity titration using 1 mM HCl) could be made. Thawed samples were analyzed for major ions (Ca^2+^, Mg^2+^, Na^+^, K^+^, Cl^-^, ${\text{NO}}_{3}^{-},$ ${\text{SO}}_{4}^{2-}$) in the UK using Dionex DX90 ion chromatography units, and for ${\text{NH}}_{4}^{+}$ using a Skalar Autoanalyzer. Precision errors were <5% according to mid-range standards for all tests.

### Data analyses

After stringent removal of technical replicates (Gomez-Alvarez et al., 2009) with cd-hit (at 99.5%; Li and Godzik, 2006), vector screening with Lucy (Chou and Holmes, 2001) and MG-RAST (Meyer et al., 2008), and eliminating shorter (<100 bp) reads, 262,086 reads (average length 441 bp) were then analyzed using MG-RAST (Meyer et al., 2008). Searches with a minimum cut-off of 1e^−05^, were made against the RDP (Cole et al., 2009), Greengenes (DeSantis et al., 2006), and SEED (Overbeek et al., 2005) databases. An alternate independent OTU analysis was carried out by screening the original set of reads for 16S sequence using both RDP and GenBank (Benson et al., 2005), eliminating redundancy, and selecting those above 90% identity. Sequences were deposited in GenBank accession number SRA060370.

## Results

### Metagenomic library

#### Preliminary analysis

End sequencing gave matches to phototrophs and halotolerant organisms such as *Nocardioides* sp., *Actinobacteria*, *Chlamydomonas reinhardtii*, *Halobacterium* sp., Halophilic archaeon, *Chromohalobacter salexigens*, *Phytophthora sojae*, and a plant pathogen, confirming that genomic DNA incorporated into the metagenomic library was predominantly derived from typical soil micro-organisms. Specific gene probing for phosphonate genes, fungal genes, and phage genes all generated successful amplifications.

#### Phylogeny from 454 pyrosequencing data

The total number of sequences containing some taxonomic information was 261,840. Blasting these sequences against the SEED database produced 322 Phyla hits (including bacteria, eukarya, viruses, and archaea). The full phylogeny of these sequences (>1,000 hits) is shown in Table 3. The numbers of genera in each class in the 454 phylogeny were (with percentage abundances in parentheses): Proteobacteria 320 genera (48.7%) as: alpha 100 genera (12.2%), beta 46 genera (4.2%), delta 27 genera (4.1%), epsilon 18 genera (0.1%), gamma 129 genera (7.5%), unclassified (0.1%), then Actinobacteria 78 genera (10.6%), Firmicutes 85 genera (7.9%), Bacteroidetes 21 genera (5.9%), Planctomycetes 3 genera (4.7%), Acidobacteria 2 genera (4.0%), Cyanobacteria 26 genera (3.4%), Verrucomicrobia 2 genera (3.1%), and Chloroflexi 9 genera (2.3%).

**Table 3:** **Phylogeny of bacterial sequences derived from 454 pyrosequencing data, with number of sequences in each group (where frequency >1,000)**.

Candidatus *Solibacter usitatus*	5,652
*Rhodopseudomonas palustris*	3,293
*Rhodopirellula baltica*	2,764
*Gemmatimonas aurantiaca*	2,735
*Sphingomonas wittichii*	2,507
Candidatus *Koribacter versatilis*	2,343
*Salmonella enterica*	2,299
*Sphingopyxis alaskensis*	2,272
*Novosphingobium aromaticivorans*	2,239
*Chthoniobacter flavus*	2,100
*Pirellula staleyi*	1,931
*Ruminococcus albus*	1,889
*Gemmata obscuriglobus*	1,811
*Planctomyces limnophilus*	1,692
*Myxococcus xanthus*	1,624
*Xylella fastidiosa*	1,601
*Bradyrhizobium japonicum*	1,571
*Verrucomicrobium spinosum*	1,519
*Opitutus terrae*	1,379
*Frankia* sp.	1,352
Bacterium Ellin514	1,341
*Bradyrhizobium* sp.	1,340
*Xanthomonas campestris*	1,332
*Erythrobacter* sp.	1,302
*Spirosoma linguale*	1,263
*Chitinophaga pinensis*	1,182
*Blastopirellula marina*	1,161
*Planctomyces maris*	1,128
*Escherichia coli*	1,127
*Synechococcus* sp.	1,120
*Erythrobacter litoralis*	1,105
*Sinorhizobium meliloti*	1,074
*Sorangium cellulosum*	1,055
*Sphingobium japonicum*	1,037
*Roseiflexus* sp. RS-1	1,019

**Figure 2:**
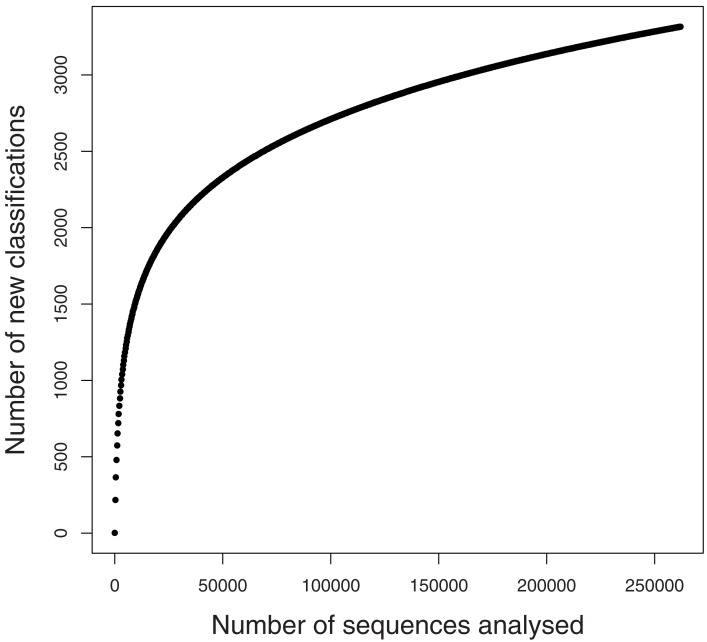
**Rarefaction graph from SEED data**.

**Figure 3:**
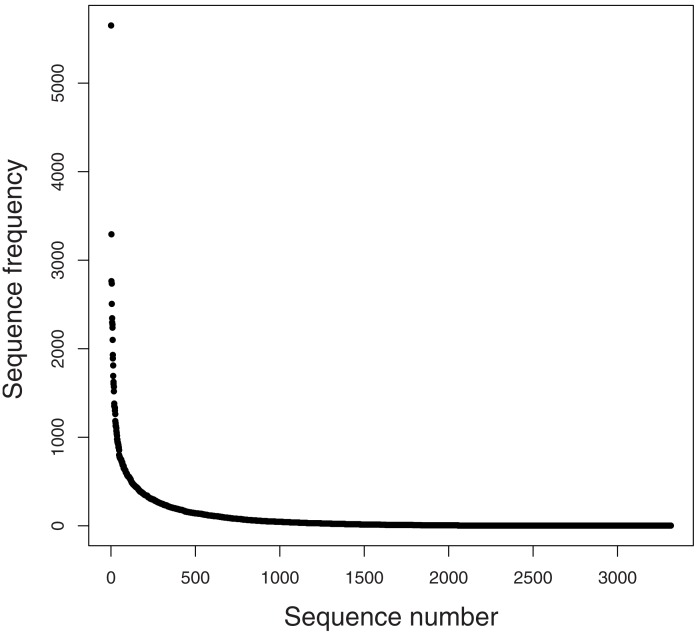
**Rare diversity graph**.

A rarefaction analysis of the final 454 data matches from the 261,840 sequences yielded 1,160 genera identified (Figure 2). An analysis of frequency distribution allowed an assessment of rare diversity, with the most common individual sequence match occurring 5,652 times (Figure 3). One hundred sixty-eight sequences occurred only once and 58 only twice. These data gave a Chao estimated sequence number of 1,400 (82.9% coverage) and a coverage estimate (Good, 1953) of 85.52%.

Of the 1,160 genera identified, the top 10 matches were to *Candidatus Solibacter* (5,652), *Burkholderia* (5,405), *Streptomyces* (5,348), *Xanthomonas* (3,685), *Pseudomonas* (3,554), *Sphingomonas* (3,432), *Planctomyces* (3,417), *Bradyrhizobium* (3,308), *Rhodopseudomonas* (3,295), and *Bacillus* (3,279). Other important groups were also present, for example, the *Methylobacteria* (2,562). Only 228 sequences had a single hit and 10,236 sequences in 73 groups were unclassified genera (3.88%).

Of the 3,316 species identified, the top 10 matches were to *Candidatus Solibacter usitatus* (5,652), *Rhodopseudomonas palustris* (3,293), *Rhodopirellula baltica* (2,764), *Gemmatimonas aurantiaca* (2,735), *Sphingomonas wittichii* (2,507), *Candidatus Koribacter versatilis* (2,343), *Salmonella enterica* (2,299), *Sphingopyxis alaskensis* (2,272), *Novosphingobium aromaticivorans* (2,239), and *Chthoniobacter flavus* (2,100). Other important groups were also identified, e.g., bacterium Ellin514 (1,341), which is commonly recorded in polar studies. Only 2,326 sequences in 328 groups were unclassified species (0.82%).

Screening the sequences using the SEED database and selecting for virus sequences gave 494 phage sequences in 28 genera (shown in parentheses). The top 10 phage type occurrences in order of frequency were *Mycobacterium* 107 (10 types), *Burkholderia* 104 (8 types), *Bordetella* 59 (3 types), *Pseudomonas* 51 (7 types), *Enterobacteria* 39 (10 types), *Flavobacterium* 22 (1 type), *Myxococcus* 14 (1 type), *Synechococcus* 11 (2 types), *Prochlorococcus* 9 (3 types), and *Sinorhizobium* 9 (1 type).

Screening sequences using the SEED database and selecting for Archaea sequences gave 32 Euryarchaeota, 16 Crenarchaeota, and 1 Korarchaeota. The top 10 Archaeal species that were recorded were *Methanosarcina acetivorans* 296 (3 types), *Methanospirillum hungatei* 77 (1 type), *Pyrococcus abyssi* 75 (3 types), *Sulfolobus acidocaldarius* 64 (3 types), *Haloarcula marismortui* 60 (1 type), *Methanococcus maripaludis* 59 (1 type), *Pyrobaculum aerophilum* 55 (4 types), *Methanoculleus marisnigri* 50 (1 type), *Archaeoglobus fulgidus* 48 (1 type), and *Methanosphaerula palustris* 48 (1 type).

Screening sequences using the SEED database and selecting for eukaryotic sequences generated few matches. These included the nematode *Caenorhabditis* sp., the liverwort *Marchantia* sp., the marine diatom *Odontella* sp., the fungi (*Gibberella* sp., *Neurospora* sp., *Magnaporthe* sp., *Schizosaccharomyces* sp., *Saccharomyces* sp., and *Eremothecium* sp.), protozoa (*Mesostigma* sp., *Naegleria* sp., and *Paramecium* sp.), and algae (*Cyanidium* sp., *Cyanidioschyzon* sp., and *Laminaria* sp.).

#### Gene ontology from 454 data

**Figure 4:**
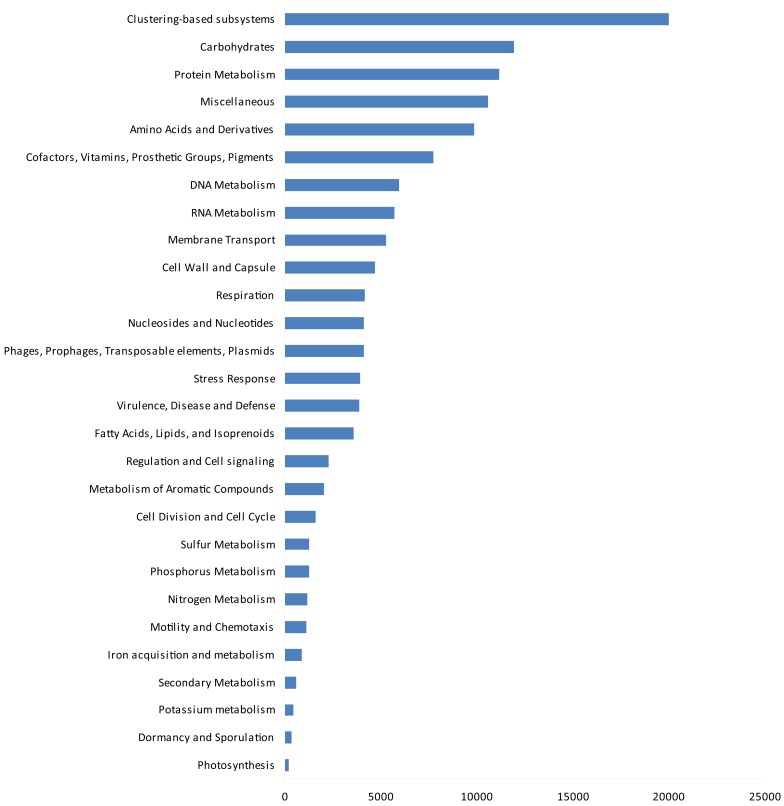
**Gene ontology by function**.

The gene ontologies derived from the metagenomic data are shown in Figure 4. Based on activity class, the most frequently encountered genes were those for clustering-based sub-systems (the precise functions of which are currently unknown), with the next most frequent genes being for carbohydrate metabolism, protein metabolism, amino acids and their derivatives, and cofactors, vitamins, prosthetic groups, and pigments. After these, the next most common genes were for DNA and RNA metabolism, membrane transport, and the cell wall and capsule. Genes for respiration, nucleosides and nucleotides, three classes of potentially ecologically important genes phages, prophages, transposable elements, plasmids, stress response genes, virulence, disease, and defense followed these. After these, four further classes of housekeeping genes (fatty acids, lipids, and isoprenoids; regulation and cell signaling, metabolism of aromatic compounds and cell division and cell cycle) occurred. Finally, ecologically important classes of genes for sulfur, phosphorus, and nitrogen metabolism, motility and chemotaxis, iron acquisition and metabolism, secondary metabolism, potassium metabolism, dormancy and sporulation, and photosynthesis were recorded.

Removing all structural, carbohydrate metabolism, and DNA/RNA processing and modification genes (77% of matches) resulted in 31 classes of protein with >500 matches (10% of matches). The most common (<6% of all matches, ∼10,000 matches) was to a protein (DUF1446) of unknown function. The next most frequent category (>1%, ∼3,000 matches) contained genes for prophage associated DNA combinational repair protein (2.4%), resistance to antibiotics and toxic compounds (2.4%), one-carbon metabolism (1.5%), and oxidative stress (1.5%). The next category, with 1,500–3,000 matches (0.5–1.0%) contained genes for CO_2_ fixation (0.86%), flagellar motility (0.7%), phosphate metabolism (0.6%), and phospholipids (0.6%). After these, 1,000–1,500 matches (0.3–0.5%) were for osmotic stress (0.45%), heat shock (0.42%), quinone cofactors (0.38%), Ton and Tol transport systems (0.38%), and ammonia assimilation (0.36%). In the next category, several groups of potentially ecologically important proteins for Antarctic soils were then recorded, 1,000–600 (0.2–0.3%) containing genes for siderophores (0.3%), high affinity phosphate transporter and control of PHO regulon (0.3%), iron acquisition (0.29%), potassium homeostasis (0.29%), organic sulfur assimilation (0.28%), nitrate and nitrite ammonification (0.28%), nucleotidyl-phosphate metabolic clusters (0.28%), inorganic sulfur assimilation (0.27%), bacterial chemotaxis (0.25%), regulation of virulence (0.24%), P uptake in cyanobacteria (0.21%), and pathogenicity islands (0.21%). Finally, <600 hits (0.15–0.2%) occurred to quorum sensing and biofilm formation (0.19%), periplasmic stress (0.19%), and detoxification genes (0.17%). A further 43,168 categories had <500 matches, with 17 classes having a single sequence associated with them.

#### Clone library re-analysis

Of 43 sequences that had earlier been grouped into 21 sequence types based on a ≥97% cut-off level, pair-wise comparison of sequences within the originally assigned groups showed that only four of these independently the criteria within the group itself (Table 4), suggesting that 14 of the original groupings underestimated total diversity.

**Table 4:** **Mars Oasis clone library re-analysis**.

Nominal identification given	Original (≥97% BLAST i.d.)	Sequence number	Sequence pair-wise comparisons (mismatch/sequence length similarity%)		
Uncultured bact clone MeCl 62	12/657 (98.2%)	3	27/841 (96.8%)	38/721 (94.7%)	33/721 (95.4%)
Uncultured eukaryote clone	36/730 (99%)	3	12/769 (98.4%)	74/802 (90.8%)	51/760 (93.3%)
Uncultured bact clone FRCH17502	3/745 (99.6%)	2	20/767 (97.4%)		
Uncultured bact clone LVH3-G7	4/715 (99%)	2	4/715 (99.0%)		
Uncultured bacteroidetes AS28	209/728 (71.3%)	2	92/725 (87.3%)		
Uncultured bacteroidetes clone	34/780 (95.6%)	3	317/826 (97.9%)	25/781 (96.8%)	29/782 (96.3%)
Uncultured cyanobacterium clone	20/699 (97.14%)	2	20/708 (97.2%)		
Uncultured Micrococcineae	64/715 (91.1%)	3	23/715 (96.8%)	48/708 (93.2%)	53/757 (93.0%)
Soil bacterial clone U8	24/795 (97.0%)	2	26/801 (96.8%)		
Actinomycetes clone FB-2 A11	28/724 (96.1%)	2	24/725 (96.7%)		
Uncultured bacterium clone CM131	32/414 (92.3%)	2	33/414 (92.0%)		
Bacterial clone KuyT-IWPB 17	26/744 (96.5%)	2	29/744 (96.1%)		
*Solirubrobacter* Gsoil 921	33/709 (95.3%)	3	7/710 (99.0%)	33/701 (95.3%)	30/767 (96.1%)
Uncultured bact 071021-ONK-KR1-12	69/812 (91.5%)	2	62/799 (92.2%)		
Uncultured bacterium FACH1766	16/805 (98.0%)	2	20/802 (97.5%)		
Uncultured bacterial clone F1-2F-F12	163/775 (79.0%)	2	80/774 (89.7%)		
Uncultured Caldilineaceae bacterium	59/734 (92.0%)	3	30/744 (96.0%)	48/761 (93.7%)	48/744 (93.5%)
*Anabaena* sp.	37/756 (92.0%)	3	18/772 (97.7%)	29/770 (96.2%)	39/780 (95.0%)

#### Chemistry

Chemical data are shown in Table 5. Runoff and soil pore water from the margin of the lake at Mars Oasis had pHs of 7.4–7.6. Sulfate and calcium were the dominant ions present in the water (2,700–4,100 μ equivalents L^−1^). Carbonate and magnesium ions were less frequent (450–1,400 μ equivalents L^−1^), followed by sodium, chloride, and potassium (13–333 μ equivalents L^−1^). Finally, ammonium-*N* and nitrate-*N* were the least frequent ions in runoff, and ammonium was infrequent in pore water (each 3–6 μ equivalents L^−1^), but nitrate was relatively frequent in the latter (200 μ equivalents L^−1^).

**Table 5:** **pH and ion concentrations of lake runoff, lake water, pore water, and snow at Mars Oasis**.

Sample	pH	${\text{NH}}_{4}^{+}$	Na^+^	K^+^	Ca^2+^	Mg^2+^	${\text{HCO}}_{3}^{-}$	Cl^-^	${\text{SO}}_{4}^{2-}$	${\text{NO}}_{3}^{-}$	O_2_	DOC
Runoff	7.6	5.61	118	12.8	2,981	784	1,392	32.6	4,082	3.33	13.0	0.35
Lake water (sample 1)	7.3	6.25	101	14.2	1,238	259	1,771	141	956	2.38	12.2	6.53
Lake water (sample 2)	7.4	4.09	129	20.0	1,540	233	1,573	108	1,310	1.80	12.9	2.43
Lake water (sample 3)	7.5	6.14	85.7	16.6	849	115	768	103	781	0.86	12.7	1.15
Lake water (sample 4)	7.7	3.11	36.8	8.37	510	64.0	1,088	26.1	165	5.92	12.6	m.d.
Pore water	7.4	2.91	124	16.2	2,693	449	652	333	2,464	199	7.31	5.59
Snow	5.7	2.03	16.5	12.0	46.1	3.91	m.d.	19.2	46.2	2.38	m.d.	3.64

*DOC, dissolved organic carbon; m.d., missing data*.

Data are expressed in μ equivalents L^-1^ except for dissolved oxygen and DOC (both mg L^-1^) and pH. Data are means of 7, 12, 3, and 4 measurements for runoff, lake water, pore water, and snow, respectively. ${\text{PO}}_{4}^{3-}$ was below detection limits of 1 μ equivalents L^−1^

## Discussion

Studies of bacterial communities from around the world suggest a wide spectrum of taxonomic diversity, from the Amazonian soils, where every sequence sampled could be different (Fierer et al., 2007) to the highly selective Rio Tinto river in Europe, with a relatively restricted biodiversity (Palacios et al., 2008). It is apparent from the current study that the bacterial community diversity in the soil at Mars Oasis lies somewhere between the extremes, with a total of 1,160 genera from 3,318 phylotypes detected in the 454 library. This is an order of magnitude greater than data from clone library studies alone, which have to date recorded 78–730 (de-replicated to 30–140) phylotypes present in Antarctic soils sampled from the sub-Antarctic Islands, the Antarctic Peninsula, and the continent itself (see references in Table 1). Of the 1,160 genera recorded in our study, 71 (6.12%) have also been identified by other studies of Antarctic soil biodiversity.

### Diversity at the genus level

The most frequent genera in Antarctic soils (identified in >3 studies) are *Rubrobacter*, *Arthrobacter*, *Oscillatoria*, *Sphingomonas*, *Chloroflexus*, *Anabaena*, *Actinobacteria*, *Microcoleus*, *Microcystis*, *Nitrosospira*, *Pseudomonas*, *Fervidobacterium*, *Xanthomonas*, *and Acidobacteria*, *Phormidium*, *Deinococcus*, *Bacteroides*, *Brevundimonas*, *Hymenobacter*, *Leptolyngbya*, *Nostoc*, *Pseudonocardia*, *Psychrobacter*, *Rhodococcus*, *Synechococcus*, *Cytophaga*, *Friedmanniella*, and *Sphingobacterium*. The former group was identified in both clone library based and 454 based studies, indicating that there is some broad agreement with previous studies, whilst the latter group was specifically identified in this study.

### Diversity at the sequence level

The 10 Antarctic soil biodiversity studies examined (based upon PCR amplification, cloning, and sequencing of 16S rRNA genes) each reported relatively low prokaryotic biodiversity in Antarctic terrestrial ecosystems. However, estimated coverage in each of these studies ranged from 30–70%, only two of the collectors’ curves approached anywhere near saturation and all were based upon the assumption that taxonomic differentiation occurs with <97% sequence similarity over 200–800 bp. Despite 97% sequence similarity being adopted for species differentiation in many biodiversity studies, it has already been demonstrated that 100% sequence similarity in the 16S rRNA gene can be found between isolates with different ecological phenotypes (Peña et al., 2010), and conversely, <97% sequence similarity has been found between isolates which have an identical ecological function. We therefore conducted a detailed re-analysis of 300 sequences (Newsham et al., 2010), and show that at a sequence similarity of 97%, only 7 of the 300 sequences were duplicated in the clone library. This re-analysis showed that the diversity of closely related sequences may have been underestimated by up to 40%. If we add to this an approximate doubling of the total number of genera identified through the addition of 454 data to the combined clone library summary, then there could be a minimum of four times greater diversity than was previously described. So although prokaryotic diversity may indeed be restricted at the generic level, there appears to be high sequence diversity present in the soil at Mars Oasis.

### Biodiversity

The range of taxa identified extended (indeed increased by five times), rather than contradicted the taxa identified in earlier clone library studies (140 Genera were present in both; 56 from clone library studies only and 1,026 from this 454 study only). A number of key taxa were identified, that have been indicated to be important in other studies of Antarctic soils – these include the Actinobacteria and Cyanobacteria. The taxa identified were also similar to those recovered in airborne samples of the Peninsula region. Hughes et al. (2004) sampled air for a 2-week period above Rothera Station on Adelaide Island, and found a range of microorganisms, including cyanobacteria, actinomycetes, diatom plastids, and other uncultivated bacterial groups. Elsewhere, on the Brunt Ice-Shelf over an isolated scientific research station, on an ice-shelf in continental Antarctica Pearce et al. (2010) found Bacilli, Pseudomonads, and Sphingomonads. In common with other environmental studies, particularly in the polar regions, many of the sequences obtained were from as yet uncultivated organisms. The detected aerial microorganisms were different from those obtained over the Antarctic Peninsula. In both aerobiological studies, a low microbial biodiversity was detected, which included many sequence replicates. In this study, there were also important genera identified that had not been described in a range of selected clone library studies (Table 6).

**Table 6:** **Diversity of bacteria (and other groups) identified to the Genus level from Antarctic soil from by 454 sequencing**.

*Abiotrophia*, *Acanthamoeba*, *Acaryochloris*, *Acetitomaculum*, *Acetivibrio*, *Acetobacter*, *Acetohalobium*, *Acholeplasma*, *Achromobacter*, *Acidaminococcus*, *Acidilobus*, *Acidiphilium*, *Acidithiobacillus*, *Acidithiomicrobium*, *Acidobacterium*, *Aciduliprofundum*, *Acorus*, *Actinocorallia*, *Actinomadura*, *Actinoplanes*, *Actinosynnema*, *Acuclavella*, *Acyrthosiphon*, *Adineta*, *Aedes*, *Aerococcus*, *Aeromonas*, *Aeropyrum*, *Afipia*, *Aggregatibacter*, *Agromyces*, *Ahrensia*, *Ailuropoda*, *Ajellomyces*, *Akkermansia*, *Albidiferax*, *Alcanivorax*, *Algoriphagus*, *Alicycliphilus*, *Alicyclobacillus*, *Aliivibrio*, *Alistipes*, *Alkalilimnicola*, *Alkaliphilus*, *Allium*, *Allochromatium*, *Alphabaculovirus*, *Alphapapillomavirus*, *Alternaria*, *Alteromonas*, *Amaranthus*, *Amatitlania*, *Aminobacterium*, *Aminomonas*, *Ammonifex*, *Amplypterus*, *Amycolatopsis*, *Anaerobaculum*, *Anaerococcus*, *Anaerofustis*, *Anaerolinea*, *Anaeromyxobacter*, *Anaerostipes*, *Anaerotruncus*, *Anaplasma*, *Ancylobacter*, *Aneurinibacillus*, *Anopheles*, *Anoplopoma*, *Anoxybacillus*, *Anthopleura*, *Antonospora*, *Aphanizomenon*, *Aphanomyces*, *Apis*, *Apteryx*, *Aquimonas*, *Arabidopsis*, *Arcanobacterium*, *Archaeoglobus*, *Arcobacter*, *Aromatoleum*, *Arsenophonus*, *Artemia*, *Arthroderma*, *Arthrospira*, *Ascidia*, *Asfivirus*, *Aspergillus*, *Asticcacaulis*, *Ateles*, *Atopobium*, *Aulacoseira*, *Aurantimonas*, *Aureobasidium*, *Aureococcus*, *Azorhizobium*, *Azospirillum*, *Babesia*, *Bacteroides*, *Basfia*, *Bathymodiolus*, *Batrachovirus*, *Beggiatoa*, *Beijerinckia*, *Bermanella*, *Beryx*, *Beutenbergia*, *Bilophila*, *Blastocystis*, *Blastomonas*, *Blastopirellula*, *Blattabacterium*, *Blautia*, *Bombyx*, *Bordetella*, *Borrelia*, *Bos*, *Botryotinia*, Bpp-1-like viruses, *Brachybacterium*, *Brachymonas*, *Brachypodium*, *Brachyspira*, *Branchiostoma*, *Brassica*, *Brevibacillus*, *Brevundimonas*, *Bromus*, *Brucella*, *Brugia*, *Bryonia*, *Bulleidia*, *Butyrivibrio*, *Caenorhabditis*, *Caldanaerobacter*, *Calditerrivibrio*, *Caldivirga*, *Caligus*, *Callithrix*, *Camelus*, *Caminibacter*, *Camponotus*, *Campylobacter*, *Canavalia*, *Candida*, Candidatus, *Accumulibacter*, Candidatus, *Amoebophilus*, Candidatus, *Azobacteroides*, Candidatus, *Blochmannia*, Candidatus, *Chloracidobacterium*, Candidatus, *Cloacamonas*, Candidatus, *Desulforudis*, Candidatus, *Hamiltonella*, Candidatus, *Korarchaeum*, Candidatus, *Koribacter*, Candidatus, *Kuenenia*, Candidatus, *Liberibacter*, Candidatus, *Magnetobacterium*, Candidatus, *Micrarchaeum*, Candidatus, *Nitrososphaera*, Candidatus, *Odyssella*, Candidatus, *Parvarchaeum*, Candidatus, *Pelagibacter*, Candidatus, *Phytoplasma*, Candidatus, *Protochlamydia*, Candidatus, *Puniceispirillum*, Candidatus, *Regiella*, Candidatus, *Rhodoluna*, Candidatus, *Solibacter*, Candidatus, *Sulcia*, *Canis*, *Capnocytophaga*, *Capsaspora*, *Capsicum*, *Carassius*, *Cardiobacterium*, *Carnobacterium*, *Catabena*, *Catenibacterium*, *Catenulispora*, *Catharanthus*, *Catonella*, *Cavia*, *Cellulomonas*, *Cellulosilyticum*, *Cellulosimicrobium*, *Cellvibrio*, *Cenarchaeum*, *Chaetoceros*, *Chaetomium*, *Chara*, *Chattonella*, *Chelativorans*, *Chitinophaga*, *Chlamydia*, *Chlamydomonas*, *Chlamydophila*, *Chlorella*, *Chlorobaculum*, *Chlorobium*, *Chloroherpeton*, *Chlorokybus*, *Chlorovirus*, *Chondrus*, *Chrysopathes*, *Chthoniobacter*, *Cicer*, *Ciona*, *Citreicella*, *Citrobacter*, *Citromicrobium Clavibacter*, *Clavispora*, *Coccidioides*, *Cochliobolus*, *Collimonas*, *Collinsella*, *Colossendeis*, *Comamonas*, *Compsopogon*, *Congregibacter*, *Coprinopsis*, *Coprobacillus*, *Coprococcus*, *Coprothermobacter*, *Coptotermes*, *Coraliomargarita*, *Corallina*, *Corynebacterium*, *Coxiella*, *Crassostrea*, *Cricetulus*, *Croceibacter*, *Crocosphaera*, *Cronobacter*, *Crustomastix*, *Crypthecodinium*, *Cryptobacterium*, *Cryptosporidium*, *Cucumis*, *Cucurbita*, *Culex*, *Culicoides*, *Cupriavidus*, *Curvibacter*, *Curvularia*, *Cyanidioschyzon*, *Cyanidium*, *Cyanobium*, *Cyanophora*, *Cylindrospermopsis*, *Cystobacter*, *Cytophaga*, *Dactylosporangium*, *Danio*, *Daphnia*, *Dasypus*, *Daucus*, *Debaryomyces*, *Deferribacter*, *Dehalogenimonas*, *Deinococcus*, *Denitrovibrio*, *Dermacentor*, *Dermacoccus*, *Desulfarculus*, *Desulfatibacillum*, *Desulfobacterium*, *Desulfobulbus*, *Desulfocella*, *Desulfococcus*, *Desulfohalobium*, *Desulfomicrobium*, *Desulfomonile*, *Desulfonatronospira*, *Desulfotomaculum*, *Desulfurispirillum*, *Desulfurivibrio*, *Desulfurococcus*, *Desulfuromonas*, *Dethiobacter*, *Dethiosulfovibrio*, *Dialister*, *Dicathais*, *Dichelobacter*, *Dickeya*, *Dictyoglomus*, *Dictyostelium*, *Dietzia*, *Discophora*, *Dokdonia*, *Dolichospermum*, *Dorea*, *Drosophila*, *Durinskia*, *Dyadobacter*, *Echinops*, *Ectocarpus*, *Edwardsiella*, *Eggerthella*, *Ehrlichia*, *Eikenella*, *Elaeis*, *Eleocharis*, *Eleotris*, *Elizabethkingia*, *Elusimicrobium*, *Elymus*, *Emericella*, *Encephalitozoon*, *Enchytraeus*, *Endoriftia*, *Enhydrobacter*, *Enhygromyxa*, *Ensifer*, *Entamoeba*, *Epiphyas*, *Epulopiscium*, *Equus*, *Eremococcus*, *Eremothecium*, *Erinaceus*, *Erwinia*, *Erysipelothrix*, *Ethanoligenens*, *Eubacterium*, *Euglena*, *Faecalibacterium*, *Felis*, *Ferrimonas*, *Ferroglobus*, *Ferroplasma*, *Filifactor*, *Filobasidiella*, *Finegoldia*, *Flammeovirga*, *Flexithrix*, *Fragilariopsis*, *Francisella*, *Fructobacillus*, *Fulvimarina*, *Fusarium*, *Fusobacterium*, *Gallionella*, *Gallus*, *Gardnerella*, *Gasterosteus*, *Gemella*, *Gemmatimonas*, *Gentiana*, *Geobacillus*, *Giardia*, *Gibberella*, *Glaciecola*, *Glomerella*, *Glossina*, *Gluconacetobacter*, *Glycine*, *Gordonia*, *Gordonibacter*, *Gorilla*, *Gracilaria*, *Gramella*, *Granulicatella*, *Grosmannia*, *Guillardia*, *Haemophilus*, *Hafnia*, *Hahella*, *Haladaptatus*, *Halalkalicoccus*, *Halanaerobium*, *Haliangium*, *Haliotis*, *Haloarcula*, *Halobacterium*, *Haloferax*, *Halogeometricum*, *Halomicrobium*, *Halomonas*, *Haloquadratum*, *Halorhabdus*, *Halorubrum*, *Haloterrigena*, *Halothermothrix*, *Halothiobacillus*, *Harpegnathos*, *Hartmannella*, *Haslea*, *Helianthus*, *Heliobacillus*, *Heliobacterium*, *Heliothis*, *Herbaspirillum*, *Herminiimonas*, *Hirschia*, *Histophilus*, *Hoeflea*, *Holdemania*, *Homo*, *Hordeum*, *Hydra*, *Hydrogenivirga*, *Hydrogenobacter*, *Hydrogenobaculum*, *Hydrogenophaga*, *Hyles*, *Hyperthermus*, *Hyphomicrobium*, *Hyphomonas*, *Ictalurus*, *Ignicoccus*, *Ignisphaera*, *Ilyobacter*, *Ipomoea*, *Isosphaera*, *Ixodes*, *Jackiella*, *Jannaschia*, *Jonesia*, *Jonquetella*, *Kalidium*, *Kangiella*, *Karenia*, *Ketogulonicigenium*, *Kineococcus*, *Kingella*, *Kitasatospora*, *Kluyveromyces*, *Kocuria*, *Kordia*, *Kosmotoga*, *Ktedonobacter*, *Kutzneria*, *Kytococcus*, L5-like viruses, *Labrenzia*, *Laccaria*, *Lachancea*, *Lactobacillus*, *Lactococcus*, Lambda-like viruses, *Laminaria*, *Lamprocystis*, *Laribacter*, *Larimichthys*, *Lautropia*, *Lawsonia*, *Leadbetterella*, *Leclercia*, *Leeuwenhoekiella*, *Legionella*, *Leishmania*, *Lentisphaera*, *Lentivirus*, *Lepeophtheirus*, *Lepidium*, *Leptolyngbya*, *Leptosphaeria*, *Leptospira*, *Leptospirillum*, *Leptothrix*, *Leptotrichia*, *Limnobacter*, *Limnoria*, *Listeria*, *Listonella*, *Loa*, *Lodderomyces*, *Lolium*, *Loxodonta*, *Lunularia*, *Lutiella*, LUZ24-like viruses, *Lymnaea*, *Lysinibacillus*, *Lysobacter*, *Lytechinus*, *Macaca*, *Macrococcus*, *Magnaporthe*, *Magnetococcus*, *Magnetospirillum*, *Malassezia*, *Malus*, *Mannheimia*, *Marchantia*, *Maribacter*, *Maricaulis*, *Marinitoga*, *Marinobacter*, *Mariprofundus*, *Maritimibacter*, *Marivirga*, *Medicago*, *Megamonas*, *Megasphaera*, *Mesembryanthemum*, *Mesorhizobium*, *Mesostigma*, *Metajapyx*, *Metallosphaera*, *Metarhizium*, *Methanobacterium*, *Methanobrevibacter*, *Methanocaldococcus*, *Methanocella*, *Methanococcoides*, *Methanococcus*, *Methanocorpusculum*, *Methanoculleus*, *Methanohalobium*, *Methanohalophilus*, *Methanoplanus*, *Methanopyrus*, *Methanoregula*, *Methanosaeta*, *Methanosarcina*, *Methanosphaera*, *Methanosphaerula*, *Methanospirillum*, *Methanothermobacter*, *Methanothermococcus*, *Methanothermus*, *Methylacidiphilum*, *Methylibium*, *Methylobacter*, *Methylobacterium*, *Methylocapsa*, *Methylocella*, *Methylocystis*, *Methylomonas*, *Methylophaga*, *Methylophilus*, *Methylosinus*, *Methylotenera*, *Methyloversatilis*, *Methylovorus*, *Metridium*, *Meyerozyma*, *Microbacterium*, *Microchaete*, *Micromonas*, *Micromonospora*, *Microtus*, *Mimivirus*, *Mitsuokella*, *Mnemiopsis*, *Mobiluncus*, *Moniliophthora*, *Monocercomonoides*, *Monodelphis*, *Monosiga*, *Moorella*, *Moraxella*, *Mucilaginibacter*, *Mus*, *Mussa*, *Mycobacterium*, *Myotis*, *Myrothecium*, *Myxococcus*, *Myzus*, N15-like viruses, N4-like viruses, *Naegleria*, *Nakamurella*, *Nakaseomyces*, *Nannocystis*, *Nanoarchaeum*, *Nasonia*, *Natranaerobius*, *Natrialba*, *Natronomonas*, *Nautilia*, *Nectria*, *Neisseria*, *Nematostella*, *Neosartorya*, *Nephroselmis*, *Neptuniibacter*, *Neurospora*, *Nicotiana*, *Nitratifractor*, *Nitratiruptor*, *Nitrobacter*, *Nitrococcus*, *Nitrosomonas*, *Nitrosopumilus*, *Nitrospira*, *Nocardiopsis*, *Nonomuraea*, *Nostoc*, *Notechis*, *Novosphingobium*, *Oceanibulbus*, *Oceanicaulis*, *Oceanithermus*, *Oceanobacillus*, *Ochrobactrum*, *Octadecabacter*, *Odontella*, *Oenococcus*, *Oenothera*, *Oikopleura*, *Okibacterium*, *Oligotropha*, *Olsenella*, *Oncorhynchus*, *Opsanus*, *Oribacterium*, *Orientia*, *Ornithobacterium*, *Ornithorhynchus*, *Oryctolagus*, *Oryza*, *Oryzias*, *Oscillochloris*, *Osmerus*, *Ostreococcus*, *Otolemur*, *Ovis*, P1-like viruses, P22-like viruses, P2-like viruses, *Paenibacillus*, *Pagrus*, *Paludibacter*, *Pan*, *Pantoea*, *Parabacteroides*, *Paracoccidioides*, *Paramecium*, *Parascardovia*, *Parvibaculum*, *Parvularcula*, *Pasteurella*, *Paucimonas*, *Paulinella*, *Pectobacterium*, *Pediculus*, *Pediococcus*, *Pelagibacter*, *Pelobacter*, *Pelodictyon*, *Pelotomaculum*, *Penicillium*, *Peperomia*, *Peptoniphilus*, *Peptostreptococcus*, *Perilla*, *Perittia*, *Perkinsus*, *Persephonella*, *Persicobacter*, *Pervagor*, *Petroselinum*, *Petrotoga*, *Pfiesteria*, *Phaeobacter*, *Phaeodactylum*, *Phaeosphaeria*, *Phascolarctobacterium*, *Phenylobacterium*, Phi29-like viruses, PhiC31-like viruses, Phieco32-like viruses, phiKMV-like viruses, phiKZ-like viruses, *Phormidium*, *Phoronis*, *Photobacterium*, *Photorhabdus*, *Physarum*, *Physcomitrella*, *Phytophthora*, *Picea*, *Pichia*, *Picrophilus*, *Picrorhiza*, *Pimelobacter*, *Pinctada*, *Pinus*, *Pisum*, *Planktothrix*, *Planobispora*, *Plasmodium*, *Plesiocystis*, *Pleurotus*, *Ploceus*, *Pneumocystis*, *Podospora*, *Poecilia*, *Polaromonas*, *Polynucleobacter*, *Polysphondylium*, *Pongo*, *Populus*, *Porphyra*, *Porphyrobacter*, *Porphyromonas*, *Postia*, *Potorous*, *Prasinovirus*, *Prauserella*, *Prionoxystus*, *Propionibacterium*, *Prosthecobacter*, *Prosthecochloris*, *Prototheca*, *Providencia*, *Pseudechis*, *Pseudendoclonium*, *Pseudonocardia*, *Pseudoramibacter*, *Pseudovibrio*, *Pseudoxanthomonas*, *Psilotum*, *Psychrobacter*, *Psychroflexus*, *Psychromonas*, *Pteris*, *Puccinia*, *Pyramidobacter*, *Pyrenophora*, *Pyrobaculum*, *Pyrococcus*, *Rahnella*, *Ralstonia*, *Rana*, *Raphidiopsis*, *Rattus*, *Reclinomonas*, *Reinekea*, *Renibacterium*, *Rhadinovirus*, *Rhinoceros*, *Rhodobacter*, *Rhodococcus*, *Rhodomonas*, *Rhodopirellula*, *Rhodopseudomonas*, *Rhodothermus*, *Ricinus*, *Rickettsia*, *Rickettsiella*, *Ricordea*, *Riemerella*, *Robiginitalea*, *Roseburia*, *Roseibium*, *Roseomonas*, *Roseovarius*, *Rothia*, *Rubritalea*, *Ruegeria*, *Ruminococcus*, *Rupicapra*, *Saccharomonospora*, *Saccharomyces*, *Saccharophagus*, *Saccharopolyspora*, *Saccharum*, *Saccoglossus*, *Sagittula*, *Salinibacter*, *Salinispora*, *Salmo*, *Sanguibacter*, *Scardovia*, *Scenedesmus*, *Scheffersomyces*, *Schistocerca*, *Schistosoma*, *Schizophyllum*, *Schizosaccharomyces*, *Sclerotinia*, *Scutigerella*, *Scytonema*, *Sebaldella*, *Segniliparus*, *Selaginella*, *Selenomonas*, *Serratia*, *Shewanella*, *Shigella*, *Shuttleworthia*, *Sideroxydans*, *Simonsiella*, *Simplexvirus*, *Sinorhizobium*, *Slackia*, *Sodalis*, *Solanum*, *Solobacterium*, *Sordaria*, *Sorex*, *Sorghum*, SP6-like viruses, SPbeta-like viruses, *Spermophilus*, *Sphaerotilus*, *Sphingobacterium*, *Sphingobium*, *Sphingopyxis*, *Spinacia*, *Spirochaeta*, *Spirogyra*, *Spirosoma*, *Spisula*, SPO1-like viruses, *Squalus*, *Stackebrandtia*, *Staphylothermus*, *Starkeya*, *Staurastrum*, *Stephos*, *Stigmatella*, *Streptoalloteichus*, *Streptobacillus*, *Streptosporangium*, *Strongylocentrotus*, *Suaeda*, *Subdoligranulum*, *Succinatimonas*, *Sulfitobacter*, *Sulfolobus*, *Sulfuricurvum*, *Sulfurihydrogenibium*, *Sulfurimonas*, *Sulfurospirillum*, *Sulfurovum*, *Sutterella*, *Synechococcus*, *Syntrophobacter*, *Syntrophomonas*, *Syntrophothermus*, *Syntrophus*, T1-like viruses, T4-like viruses, T7-like viruses, *Taenia*, *Taeniopygia*, *Takifugu*, *Talaromyces*, *Teredinibacter*, *Terracoccus*, *Terriglobus*, *Terrimonas*, *Tetrahymena*, *Tetraodon*, *Thalassiosira*, *Thalassobium*, *Thauera*, *Theileria*, *Thermaerobacter*, *Thermanaerovibrio*, *Thermincola*, *Thermoactinomyces*, *Thermoanaerobacter*, *Thermoanaerobacterium*, *Thermobaculum*, *Thermobispora*, *Thermococcus*, *Thermocrinis*, *Thermodesulfovibrio*, *Thermofilum*, *Thermomicrobium*, *Thermomonospora*, *Thermoplasma*, *Thermoproteus*, *Thermosediminibacter*, *Thermosinus*, *Thermosphaera*, *Thermosynechococcus*, *Thermovibrio*, *Thioalkalivibrio*, *Thiobacillus*, *Thiocapsa*, *Thiococcus*, *Thiorhodovibrio*, *Thylacodes*, *Tolumonas*, *Tolypothrix*, *Toxoplasma*, *Toxoptera*, *Trabulsiella*, *Tribolium*, *Trichinella*, *Trichocolea*, *Trichomonas*, *Trichophyton*, *Trichoplax*, *Triglochin*, *Triticum*, *Truepera*, *Trypanosoma*, *Tsukamurella*, *Tuber*, *Tubularia*, *Tupaia*, *Turbo*, *Turicibacter*, *Uncinocarpus*, *Ustilago*, *Vanderwaltozyma*, *Varicellovirus*, *Variovorax*, *Veillonella*, *Verrucomicrobium*, *Verticillium*, *Vicia*, *Victivallis*, *Vigna*, *Vitis*, *Volvox*, VP2-like phages, *Vulcanisaeta*, *Waddlia*, *Weeksella*, *Weissella*, *Wigglesworthia*, *Wolbachia*, *Wolinella*, *Xanthobacter*, *Xenopus*, *Xenorhabdus*, *Xylanimonas*, *Xylaria*, *Yarrowia*, *Yersinia*, *Zea*, *Zingiber*, *Zoophthora*, *Zunongwangia*, *Zygnema*, *Zygosaccharomyces*, *Zymomonas*

### Gene ontology

The gene ontology data in the present study, when expressed by activity class, gave some potential insights into the presence of functional genes in the soil at Mars Oasis. Although many genes for clustering-based sub-systems were encountered in the 454 library, suggesting the functional coupling of genes whose present purpose is unknown, the data clearly indicated that the microbial community was active, with the presence of many genes for cell division and the cell cycle, cell wall formation, nucleotides and nucleosides, and RNA metabolism. This indicates that there is potential for the soil community at the oasis to express these genes, at least during the austral summer, when temperatures are typically above freezing point during the daytime and liquid water is freely available. There is also the potential for active competition between microbes in the soil, with the presence of antibiotic and toxic compound resistance genes, quorum sensing, and biofilm formation genes and many genes relating to virulence. Given the abundance of *Actinobacteria* in the soil, and particularly genera such as *Streptomyces*, which are active synthesizers of antibiotics, it is unsurprising that many virulence genes were encountered in the soil. Genes found at lower frequencies than expected were those for stress responses (including oxidative stress, osmotic stress, periplasmic stress, cold shock, and detoxification genes), perhaps reflecting the not unfavorable environmental conditions for soil microbial growth at Mars Oasis during the summer, and those for photosynthesis. Given the abundance of Cyanobacteria in the lake margin at Mars Oasis (Wynn-Williams, 1996), it was surprising that only ∼1% of the genes in the library encoded for CO_2_ fixation. Although genes for nitrogen cycling expressed by phyla such as *Acidobacteria* were not found in the library, the use of probes indicated the presence of using *nifH*, *nosZ*, and *nirS* genes in the soil (data not shown). Genes for sulfur, phosphorus, and nitrogen metabolism were all present at about 1%, whilst those for iron acquisition and metabolism were 0.7% and potassium metabolism 0.3%.

The gene ontology data, if expressed as actual function, corroborate the view that the community at Mars Oasis is active during summer, with the potential expression of many genes for cytoskeleton and ribosome formation. The presence of genes for the utilization of lactose and galactose indicate that the microbes in the soil most probably utilize relatively simple sugars for growth: there are few plants at the oasis, and those that are present are bryophytes, which typically do not form complex aromatic molecules such as lignin. Nevertheless, some capacity within the microbial community was found for the assimilation of aromatic compounds, with the presence of genes for the assimilation of peptides, which are known to be of importance to the nitrogen cycle in soils of the northern Maritime Antarctic (Hill et al., 2011).

### Soil chemistry

The dominant ion in runoff and soil pore water was sulfate, which is almost certainly derived from the oxidation of sulfide minerals in the local shales. Like the carbonate sources, there were also clear signs of secondary minerals (gypsum and/or anhydrite) contributing to the high ${\text{SO}}_{\text{4}}^{\text{2}-}$ concentrations. Furthermore, carbonate precipitates were visible around the base of all larger clasts in the soils (Andre and Hall, 2004), and so dissolution of secondary carbonates will have contributed to the high concentrations of Ca^2+^ and ${\text{HCO}}_{3}^{-}$ also present in waters. The major ion geochemistry of surface waters at Mars Oasis therefore seems to be controlled by reactive carbonate and sulfide mineral phases and the precipitation of secondary salts following the evaporation of sediment pore waters, with coupled pyrite oxidation and carbonate dissolution, and carbonate and anhydrite dissolution and precipitation dominating the rock weathering reactions.

## Summary

Studies during the twentieth century suggested that Antarctic soils are of comparatively low microbial biodiversity (Wynn-Williams, 1996). This is certainly true when most Antarctic soils are compared to temperate or tropical soils (Fierer and Jackson, 2006; Fierer et al., 2007). It appears from the current study that prokaryotic diversity in soil at Mars Oasis is limited at the generic level, with the frequent occurrence of *Actinobacteria* and Cyanobacteria. However, at the sequence level, there appears to be much greater heterogeneity than was previously thought, perhaps owing to high divergence within the relatively restricted lineages that have successfully colonized Antarctic terrestrial environments. However, the process of grouping sequences can have an impact. Furthermore, by grouping the sequences based upon genera-level identification, you do lose the distinction of possible species and strain level diversity. As more studies on the microbial diversity present in Antarctic soil using molecular techniques become available, particularly those using mass sequencing on soils sampled from transect studies along the Antarctic Peninsula, it will become clear whether Antarctic terrestrial prokaryotic diversity is higher than was originally thought, and whether potential biodiversity hot spots, such as Mars Oasis, occur in this region (Yergeau et al., 2007).

## Conflict of Interest Statement

The authors declare that the research was conducted in the absence of any commercial or financial relationships that could be construed as a potential conflict of interest.
